# Mental health dynamics between mothers and siblings of children with disabilities

**DOI:** 10.3389/fpsyg.2024.1501343

**Published:** 2024-12-24

**Authors:** Erica Zahl, Hugo Cogo-Moreira, Trude Fredriksen, Solveig Kirchhofer, Stian Orm, Torun Vatne, Matteo Botta, Krister Fjermestad

**Affiliations:** ^1^Department of Psychology, University of Oslo, Oslo, Norway; ^2^Østfold University College Faculty of Education, Fredrikstad, Norway; ^3^Innlandet Hospital Trust, Brumunddal, Norway; ^4^Nic Waals Institute, Child and Adolescent Mental Health Services, Lovisenberg Hospital Trust, Oslo, Norway; ^5^Department of Psychology, Inland Norway University of Applied Sciences, Lillehammer, Norway; ^6^Frambu Resource Centre for Rare Disorders, Siggerud, Norway

**Keywords:** disability, parental mental health, sibling mental health, sibling adjustment, familial mental health dynamics

## Abstract

**Introduction:**

When a child has a disability, their families face significant challenges that also impact parents’ and siblings’ mental health and adjustment. We examined the potential bidirectional relationships between parental mental health and sibling mental health and adjustment in families of children with a disability.

**Methods:**

We utilized baseline and 12-month follow-up data from a randomized controlled trial of a brief intervention designed to enhance parent-sibling communication in families of children with a disability. The sample comprised 214 siblings aged 8–16 years and their parents (*N* = 203 mothers, *N* = 124 fathers). We estimated bivariate latent change score models to examine the longitudinal cross-domain associations between changes in parental mental health and changes in sibling mental health, and changes in parental mental health and sibling adjustment.

**Results:**

The results showed that changes in maternal mental health and sibling adjustment over the 12-month period were correlated (*r* = 0.22). The cross-domain associations between changes in maternal mental health and sibling mental health and adjustment were not statistically significant in any of the two models. However, baseline sibling mental health had nearly doubled impact on changes in maternal mental health (*Β* = 0.232, *p* = 0.061) compared to maternal health’s impact on sibling mental health (*Β* = −0.134, *p* = 0.289). Models with paternal mental health data unfortunately did not run due to low paternal response rate at 12-months.

**Discussion:**

The findings suggest that whereas maternal mental health and sibling adjustment changes are correlated over time, the relationship between maternal and sibling measures does not necessarily operate bidirectionally. Future studies on family mental health dynamics should include data from fathers that may contribute to a broader understanding of these complex relationships.

## Introduction

Children with a disability may face difficulties with communication, mobility, intellectual and/or social functioning (World Health Organization, 2011). A disability can impact children’s inclusion and engagement in typical childhood activities and environments (Anaby et al., 2013). Herein, “disability” refers difficulties in functioning due to an enduring developmental, physical, or psychological diagnosis that is either congenital or occurs during childhood. Having a child with a disability in the family poses increased risk for challenges related to family health (Kuhlthau et al., 2022). Children with a disability often require extended levels of care and specialized support (World Health Organization, 2014), which may impact family functioning in several ways. Studies have shown that both parents and siblings of children with a disability experience heightened levels of mental health challenges compared to the general population (Gau et al., 2012; Wolff et al., 2023).

Parenting a child with a disability can pose significant challenges to parental mental health through various stressors. Parents often face an increased caregiving burden, heightened stress from financial strain, the demands of frequent medical appointments, and ongoing uncertainty about their child’s condition (Pinquart, 2018; Scherer et al., 2019; Wollney et al., 2023). Identified risk factors for parental mental health include the severity of the child’s diagnosis and household income, with higher levels of mental health challenges associated with more severe diagnoses and lower-income households (Scherer et al., 2019). Additionally, the impact of caregiving demands varies by the parent’s role, as some studies show that mothers tend to report higher levels of mental health challenges compared to fathers, suggesting gender differences in response to the enhanced caregiving responsibilities (Pinquart, 2018; Zahl et al., 2024). An important concern from a family perspective is that poor parental mental health is a well-known risk factor for child mental health (Engelhard et al., 2022).

Having a sibling with a disability is constitutes a heightened risk for mental health challenges. Siblings of children with a disability are more likely to be diagnosed with depression or other mental health challenges than siblings of children without disabilities (Marquis et al., 2019), and tend to report higher mental health rating scale scores than comparison groups (Martinez et al., 2022). Siblings of children with disabilities often face emotional strain from differential parental attention, communication difficulties, and reduced predictability and social support (Haukeland et al., 2022; Incledon et al., 2015; Kirchhofer et al., 2022; Stoneman, 2001), all of which can impact their mental health negatively. Many cope with these complex feelings on their own, often suppressing their own needs to avoid adding to their parents’ stress. For instance, studies show siblings tend to conceal rather than openly share their emotions with parents (Haukeland et al., 2015).

It is important to examine the interplay between parent and sibling mental health in these family dynamics. This is because previous research typically has focused on the individual challenges faced by parents and siblings (e.g., Kelada et al., 2022; Pinquart, 2018). Hence, a gap remains in understanding the potentially bidirectional influences between parent and sibling mental health. Understanding the relationship between the mental health of parents and healthy siblings in families facing a childhood disability is crucial for providing appropriate support and intervention strategies; enhanced knowledge of these dynamics can improve family-centered care practices. Widely regarded as the gold standard in pediatric care, family-centered care is believed to lead to better health outcomes, wiser resource allocation, and greater patient and family satisfaction (Pettoello-Mantovani et al., 2009).

Studies of bidirectionality in general population samples have found that effects of child mental health on parent mental health appear stronger than the opposite effect of parent mental health on child mental health. One study found a bidirectional relationship between maternal and adolescent depression, where the adolescent-to-mother effect was stronger than the mother-to-adolescent effect. In the father-adolescent dyads, only the effect from adolescent to father was significant (Hou et al., 2020). Another study found a significant adolescent-to-mother effect that was twice as strong as the non-significant mother-to-adolescent effect, while no longitudinal effect was found between adolescents and fathers (Schulz et al., 2021). A third study found no bidirectional relationship between parent and child mental health (Larrucea-Iruretagoyena and Orue, 2023). To our knowledge, potential bidirectionality between parents and healthy siblings in families of children with a disability has not been explored previously. Additionally, while previous studies have used cross-lagged models, our study applies a bivariate latent change score model, enabling us to explore whether parents or siblings act as primary drivers of change in mental health in families of children with disability.

The transactional model of development (Sameroff, 2009) emphasizes the bidirectional nature of familial relationships and the reciprocal influence of individual behaviors, emotions, and cognitions within the family system. The transactional model posits that development is not a unidirectional process but instead emerges from continuous, reciprocal interactions between individuals and their environment over time (Sameroff, 2009). Rather than viewing family members’ roles in isolation, the model suggests that each member’s behaviors, emotions, and psychological states actively shape and are shaped by others within the family system. In the context of families with children with disabilities, the transactional model suggests that mental health outcomes among family members are not merely the result of individual stressors or dispositions, but are embedded in the dynamic familial interplay. The model emphasizes the relevance of investigating the sibling driven effect in familial mental health dynamics as it stresses children’s impact on their context over time (Sameroff, 2009). As parents are instinctively concerned about their children’s wellbeing, it is plausible that worsened mental health in the healthy sibling would emotionally activate parents stronger than how parental functioning would affect children.

The current study aims to contribute to the growing body of knowledge about family mental health dynamics by examining the bidirectional influence of parent and sibling mental health in families of children with disability. Within family-centered care, understanding the drivers of change in familial mental health dynamics is beneficial to target interventions in the most effective way. Grounded in the transactional model (Sameroff, 2009), we seek to investigate the extent to which change in parent and/or sibling mental health or adjustment over the course of 12 months is a function of the baseline levels in both or one of the family members. When investigating mental health dynamics in families of children with a disability, it is advantageous to utilize a measure for siblings that captures central features of their family situation, as well as measures that allows for comparison with children in more typical family situations. Therefore, we include both a general measure of sibling mental health and specific measure of sibling adjustment. To examine how these two sibling measures relate to one another, we initially explore the extent to which change in either adjustment or mental health in siblings is a function of the baseline level of both or one of the domains.

We examine the following research questions (RQs): RQ1: Does baseline sibling mental health predict changes in sibling adjustment and does baseline sibling adjustment predict changes in sibling mental health? RQ2: Does baseline parental mental health predict changes in sibling mental health, and does baseline sibling mental health predict changes in parental mental health? RQ3: Does baseline parental mental health predict changes in sibling adjustment and does baseline sibling adjustment predict changes in parental mental health? RQ4: Are changes in parental mental health associated with changes in sibling mental health and/or adjustment over time?

We explored the RQs openly rather than providing specific hypotheses. This is because previous studies on bidirectionality in parent and child mental health have been inconclusive (Hou et al., 2020; Larrucea-Iruretagoyena and Orue, 2023; Schulz et al., 2021). Moreover, both strengthened and weakened bidirectional relationships are plausible outcomes in families of children with disability: Given that both parents and siblings of children with a disability may experience more risk factors for poor mental health compared to families of children without disability, one may suspect that the relationships between the individual challenges may be stronger in families of children with a disability. Conversely, parents and siblings are likely highly impacted by potentially different specific circumstances related to their family member with a disability, such as heightened levels of stress and worry for parents versus jealousy or stigma for siblings. Such differences may reduce the bidirectional effect parents and siblings have on each other.

## Methods

### Participants and procedures

Siblings and their mothers and fathers were recruited for a cluster randomized controlled trial (C-RCT) of an intervention aimed at enhancing sibling mental health and family communication. Findings from the main trial showed non-significant small effect size differences in the desired direction for intervention versus waitlist at 3-months (Kirchhofer et al., 2024). Families in the waitlist arm received intervention after the 3-month effect comparator timepoint. In the current study, we consider data from baseline to the 12-month follow-up, hence an endpoint at which all participants had received intervention. Follow-up data for both the intervention and control arms were collected 12 months after the initial intervention, ensuring a consistent 12-month interval between intervention and follow-up across both groups. The main purpose of the current study was to explore bidirectional change regardless of the randomization assignment. Participants were recruited through primary and specialist health care services, advertisements, user organizations, and specialist centers. The intervention was free of charge and was delivered as part of ordinary health care. The inclusion criteria for participating siblings were (1) being the sibling of a child diagnosed with a disability aged 0–18 years, receiving specialist and/or municipal health services; (2) age 8–16 years; and (3) one parent able to attend the intervention (Fjermestad et al., 2020). Participants with complete mother and sibling mental health and adjustment data at baseline were included in the current study (*N* = 214). The C-RCT was preregistered at clinicaltrials.gov: (10.1186/s13063-020-04781-6).

The participating siblings (*N* = 214; 57.3% female, 42.7% male) had a mean age at baseline of 10.4 years (range 8–16 years, SD = 2.0). Mothers had an average age of 42.1 years (range 28–54 years; SD = 5.4). Further demographic information is presented in Table 1. Each participating family also had a child between the ages of 0 and 18 years diagnosed with a disability. The intervention was provided at clinics located in both rural and urban areas across Norway. Prior to data collection, parents provided written informed consent for themselves and on behalf of children under the age of 16 years. Siblings and parents were informed that involvement was voluntary and about the right to withdraw from the study at any time. The Regional Committee for Medical Research Ethics South East Norway (REK South East) approved the study (#2018/2461).

**Table 1:** Sample demographic information.

Baseline characteristics	*N* (%)
Participating siblings	
Female	121 (57.3)
Male	90 (42.7)
Ethnicity	
Europe	184 (87.2)
Asia	3 (1.4)
Africa	3 (1.4)
Mixed	10 (4.7)
Mother education level	
Lower secondary	6 (2.8)
Upper secondary	34 (16.1)
University (4 years)	65 (30.8)
University (>4 years)	95 (45)
Child with disability	
Male	130 (61.6)
Female	70 (33.2)
Mean age (range, SD)	10.4 (3–18, SD = 3.2)
Attention deficit hyperactivity disorder	68 (32.2)
Tourette syndrome	21 (10)
Asperger syndrome	31 (14.7)
Autism	32 (15,2)
Specific developmental disorder	2 (0.7)
Emotional or conduct disorder	9 (3.1)
Eating disorders	13 (4.5)
Cerebral palsy	7 (2.4)
Intellectual disability	8 (2.8)
Down syndrome	29 (10)
Rare disorders	53 (18.3)
Somatic disorders	3 (1)

Totals do not equal *N* = 214 due to missing responses Diagnoses were reported by parents and cross-checked with clinic registry.

### Measures

#### Parental mental health

The 90-item Symptom Checklist (SCL-90) (Derogatis, 2010) was used to measure parental mental health. All items were answered on a 5-point Likert scale (0 = *Not at all*, 1 = *A little*, 2 = *Moderate*, 4 = *Quite a lot*, 4 = *Very much*), reflecting the extent of corresponding symptoms experienced during the previous week. Examples of items include: “Crying easily” and “Suddenly scared for no reason.” The global General Symptom Index (GSI), the mean of all 90 items, was utilized as measure of parental mental health in the current study. Good reliability has been demonstrated for all subscales, with Cronbach’s alphas between 0.79 and 0.89 (Siqveland et al., 2016). The reliability of the SCL-90 in the current study was also good, with Cronbach’s alphas of 0.97 for mother GSI. To evaluate the level of SCL-90 scores in our sample, we used the Norwegian norms presented by Derogatis (2010).

#### Sibling mental health

The Strengths and Difficulties Questionnaire (SDQ) (Goodman, 2001) was used to assess siblings’ mental health. The SDQ self-report consists of 25 attributes, both positive and negative, rated on a 3-point Likert scale (0 = Not true, 1 = Somewhat true, 2 = Certainly true) to indicate how accurately each attribute has applied to the respondent over the past 6 months. Example items include: “I am often unhappy, depressed or tearful” and “I worry a lot.” The items are organized into five subscales, each containing five items: emotional symptoms, conduct problems, hyperactivity/inattention, peer problems, and prosocial behavior. The first four subscales are summed to calculate a total difficulties score, which served as the measure of sibling mental health in the current study.

The total difficulties score (Goodman, 1997), has demonstrated satisfactory reliability, with a Cronbach’s alpha of 0.80 for self-reported measures (Goodman, 2001). Furthermore, several studies have demonstrated stronger psychometric properties of the total difficulties score compared to its subscales (e.g., Ortuño-Sierra et al., 2022; Theunissen et al., 2022). In the present study, the reliability of the total difficulties score was also satisfactory, with a Cronbach’s alpha of 0.77. The validity of the SDQ has been established through its strong associations between all subscales and the presence or absence of psychiatric disorders; all scales are associated with relevant *DSM-IV* diagnoses (Goodman, 2001). High scores have been shown to be associated with a substantial increase in psychiatric risk. To evaluate the level of the SDQ scores in our sample, we compared them to means from a Dutch general population sample (Vugteveen et al., 2022).

#### Sibling adjustment

The Negative Adjustment Scale (NAS) (Lobato and Kao, 2002) was used to measure sibling adjustment. The 16-item composite score of the NAS (Orm et al., 2021) covering difficult interpersonal and intrapersonal experiences related to the sibling and family situation, was utilized as measure of sibling adjustment. All items were answered by siblings on a 4-point Likert scale (1 = *Never*, 2 = *A little*, 3 = *Sometimes*, 4 = *A lot*). Examples of items include “I feel sad because of my brother or sister’s problem,” and “My brother or sister’s problem changes what we can do as a family.” Satisfactory reliability has been demonstrated for the NAS in previous studies, with Cronbach’s alpha of 0.69–0.79 (Haukeland et al., 2022; Lobato and Kao, 2002). In the current study, the NAS also demonstrated satisfactory reliability, with Cronbach’s alpha of 0.79. To evaluate the level of the NAS scores in our sample, we compared to findings reported in Lobato and Kao (2002).

### Data analytic plan

Three bivariate latent change score (BLCS) models were estimated using Mplus version 8.7 (Muthén and Muthén, 2017). BLCS models allow investigation of the concept known as cross-domain coupling, meaning the extent to which change in one domain is a function of the baseline level in the other (Kievit et al., 2018). In the first model, to establish the relationship between the two sibling measures, we explored the extent to which change in mental health was a function of the baseline level of adjustment and the extent to which change in adjustment was a function of the baseline level of mental health. In the second model, we explored the extent to which change in mother mental health was a function of the baseline level of sibling mental health, and the extent to which change in sibling mental health was a function of the baseline level of mother mental health, was explored. In the third model, a cross-domain relationship between mother mental health and sibling adjustment was explored. Potential bidirectional influences are investigated by BLCS models, while individual change processes and possible correlations in changes across the two domains are also accounted for Kievit et al. (2018).

A BLCS model with two time points is a just-identified model, meaning the degrees of freedom is equal to zero (Ramlall, 2016). Therefore, inspecting model fit indices (i.e., root mean square of error approximation, comparative fit index, Tucker-Lewis index) is not meaningful. Theoretical details regarding this model are described by McArdle and Nesselroade (2014) and Steyer et al. (1997), and different R syntaxes as illustrations are found in Kievit et al. (2018).

**Figure 1:**
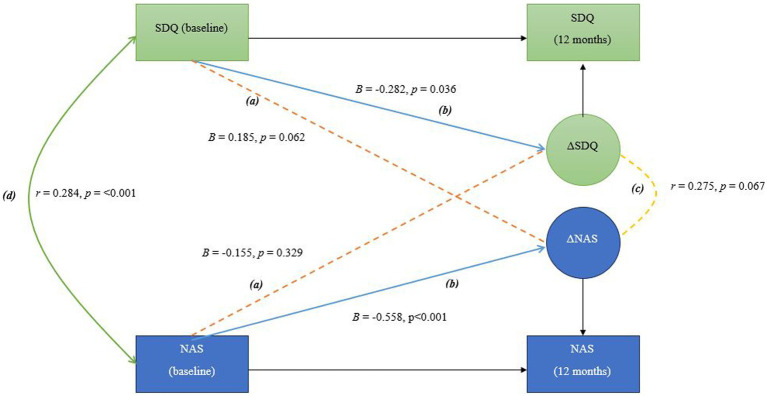
In this bivariate latent change score model, the following pathways are interpreted as follows: **(A)** Longitudinal cross-domain associations (shown in orange) represent the influence of baseline sibling mental health on the change in sibling adjustment over time, and the influence of baseline sibling adjustment on the change in sibling mental health over time **(B)** Self-feedback (shown in blue) reflects the effect of each variable’s baseline level on its own degree of change; **(C)** Correlated change (shown in yellow) shows the extent to which changes in the two variables are associated over time; **(D)** Covariance at baseline (shown in green) indicates the initial relationship between the two variables before any changes occur, representing their baseline association.

**Figure 2:**
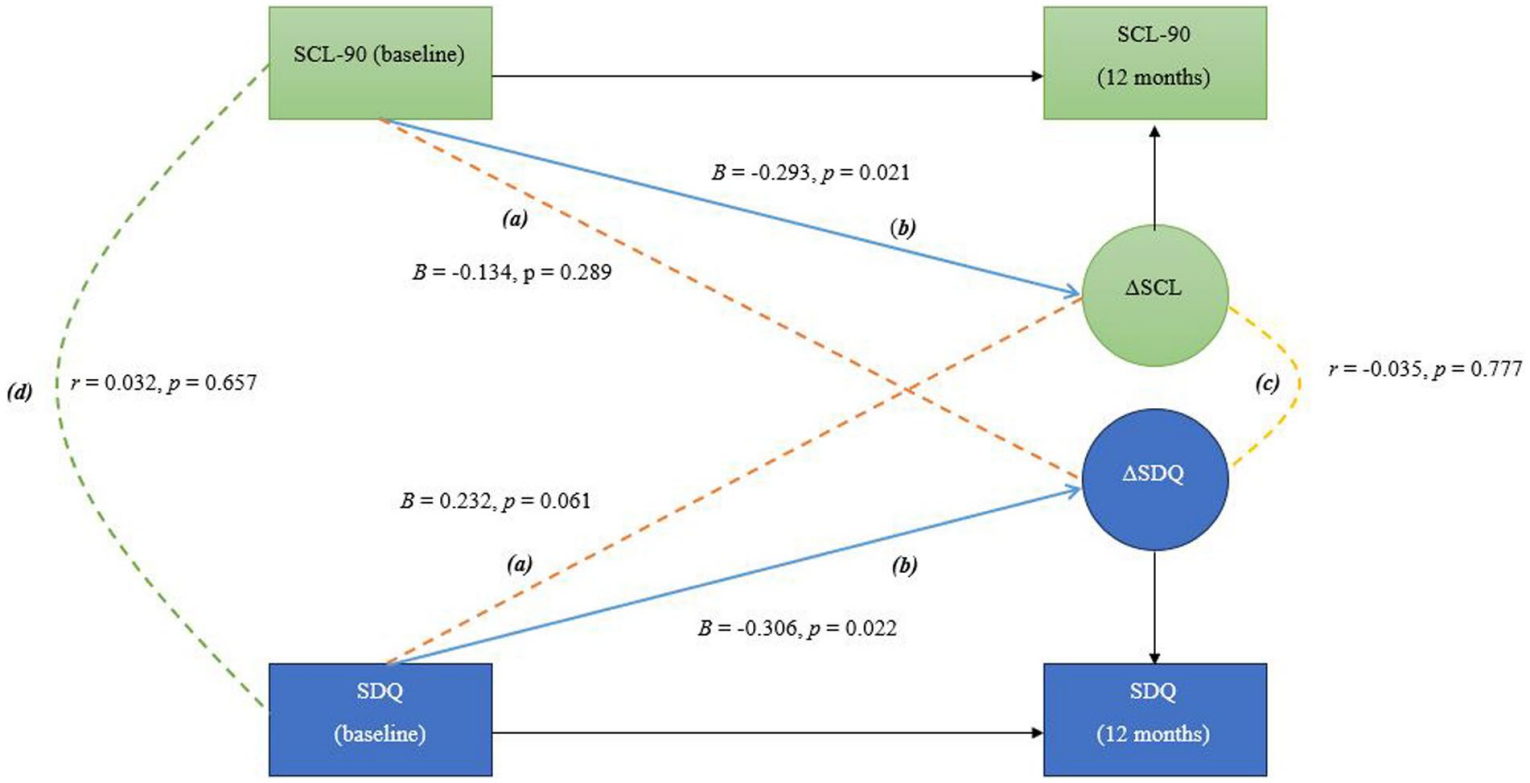
In this bivariate latent change score model, the following pathways are interpreted as follows: **(A)** Longitudinal cross-domain associations (shown in orange) represent the influence of baseline mother mental health on the change in sibling mental health over time, and the influence of baseline sibling mental health on the change in mother mental health over time **(B)** Self-feedback (shown in blue) reflects the effect of each variable’s baseline level on its own degree of change; **(C)** Correlated change (shown in yellow) shows the extent to which changes in the two variables are associated over time; **(D)** Covariance at baseline (shown in green) indicates the initial relationship between the two variables before any changes occur, representing their baseline association.

**Figure 3:**
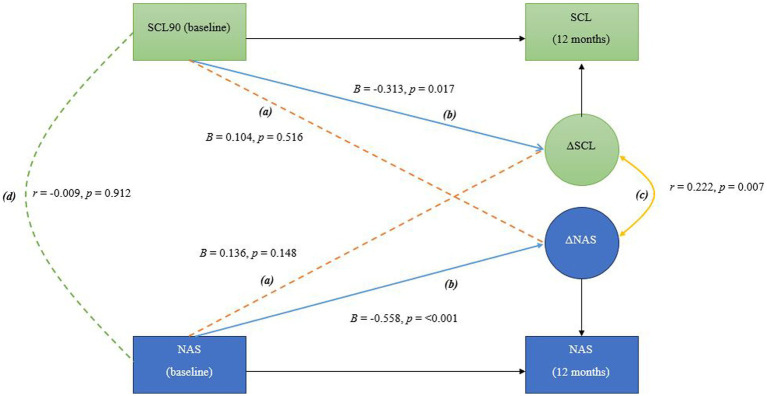
In this bivariate latent change score model, the following pathways are interpreted as follows: **(A)** Longitudinal cross-domain associations (shown in orange) represent the influence of baseline mother mental health on the change in sibling adjustment over time, and the influence of baseline sibling adjustment on the change in mother mental health over time **(B)** Self-feedback (shown in blue) reflects the effect of each variable’s baseline level on its own degree of change; **(C)** Correlated change (shown in yellow) shows the extent to which changes in the two variables are associated over time; **(D)** Covariance at baseline (shown in green) indicates the initial relationship between the two variables before any changes occur, representing their baseline association.

Using baseline and 12-month data, the three models were estimated to explore the following: (a) longitudinal cross-domain associations (shown as (a)/in orange in Figures 1–3) between baseline levels and changes in sibling mental health and adjustment (RQ1), between parental mental health and changes in sibling mental health and adjustment, as well as between baseline levels of sibling mental health and adjustment and changes in parental mental health (RQ 2 and 3), (b) self-feedback, meaning the relative individual change, of each domain (shown as (b)/in blue in Figures 1–3), (c) the correlated change (shown as (c)/in yellow in Figures 1–3), reflecting the degree to which changes in mother and sibling measures co-occur after taking into account the coupling pathway (RQ 4), and (d) covariance between measures at baseline (shown as (d)/in green in Figures 1–3).

Because this study is based on data from a cluster randomized trial (Fjermestad et al., 2020). All the analyses were conducted using maximum likelihood with robust standard errors (MLR), which deals with the non-independence of the observation (i.e., families nested in different intervention groups); then, the robust standard errors were computed considering such multilevel structure by the command in Mplus called (TYPE = Complex) as proposed by Asparouhov (2005), using a sandwich estimator (Asparouhov, 2006; Asparouhov, 2005). Missing data were dealt with full-information maximum likelihood approach under the missing at random mechanism (Enders and Bandalos, 2001). Note that maximum likelihood estimation (i.e., full-information maximum likelihood) in incomplete data analyses is based on the assumption of data missing at random (MAR), where the probability of missingness is unrelated to the actual missing values (e.g., Little and Rubin, 2002). However, MAR is not statistically testable. The structure of the BLCS model includes baseline assessments of the pairs of outcomes of interest, which serve as the primary predictors of follow-up measures and auxiliary variables (AVs) in the analysis of incomplete data sets. Because baseline and follow-up measures are at least moderately correlated, this enhances the plausibility of the MAR assumption. In other words, including baseline measures in the analysis predominantly serves to strengthen the MAR assumption’s plausibility and potentially increase statistical power, resulting in analyses that approximate MAR and facilitate trustworthy interpretation of maximum likelihood estimates (Enders, 2010). The association between a set of covariates (sibling sex, sibling and mother age, mother education level, mother employment status, plus all included diagnoses) and a dichotomous variable representing data completeness for mothers and siblings at 12 months was analyzed using logistic regression. Results are presented in Supplementary Tables.

Figures 1–3 show the main parameters of interest in their standardized form (i.e., standardized regression coefficients and Pearson’s correlations). The adopted significance level was 0.05 and the significant paths were marked as solid lines to ease the visualization.

Data and code are available upon request to the corresponding author.

## Results

Due to an extremely low response rate from fathers at 12-month follow-up (only 26 fathers reported their mental health at 12 months), there was not enough covariance coverage to run the models using fathers’ data and hence only mother-sibling dyads were included in analyses. The descriptive statistics of all included measures are presented in Table 2. The mean level of the mother mental health scores in our sample at baseline was higher than the normative SCL-90 GSI score among Norwegian women, indicating a greater degree of psychological distress for mothers in our sample. A previous study of the same sample as the current study found that both mothers and fathers in our sample had significantly higher scores on the SCL-90 compared to established norms (Zahl et al., 2024). The study however also showed that mothers scored significantly higher than fathers on the GSI and all subscales of the SCL-90. [For comprehensive comparative analyses of mothers and fathers in our sample, see Zahl et al. (2024)]. Our sample’s baseline mean level on sibling mental health was slightly elevated compared to mean values in a general population sample (Vugteveen et al., 2022), suggesting that children in our sample might face more challenges compared to peers. Our sample’s NAS mean scores were similar to those from the study conducted by Lobato and Kao (2002) examining siblings of children with a disability.

**Table 2:** Univariate high-order descriptive statistics of all measures.

Measure	Time	*N*	Mean	Variance	Skewness	Kurtosis
SCL-90	Baseline	203	0.62	0.190	0.698	0.289
	12 months	61	0.61	0.256	1.447	2.467
SDQ	Baseline	214	9.82	29.140	0.670	0.431
	12 months	44	10.0	35.931	1.106	1.001
NAS	Baseline	189	34.3	53.818	0.289	−0.164
	12 months	43	30.9	44.111	−0.425	1.001

SCL-90, Symptom Checklist 90; SDQ, strengths and difficulties questionnaire; NAS, negative adjustment scale.

*RQ1: Does baseline sibling mental health predict changes in sibling adjustment and does baseline sibling adjustment predict changes in sibling mental health?* The BLCS for sibling mental health and adjustment is shown in Figure 1. Longitudinal cross-domain associations between sibling mental health and adjustment were not statistically significant, only the self-feedback parameters were statistically significant in this sibling model. There was a significant covariance between the two sibling measures at baseline. There was not a significant correlation between the degree of change in sibling measures.

*RQ2: Does baseline parental mental health predict changes in sibling mental health, and does baseline sibling mental health predict changes in parental mental health?* The BLCS for mother mental health and sibling mental health is shown in Figure 2. Longitudinal cross-domain associations between mother mental health and sibling mental health were not statistically significant. Only the self-feedback in the two domains were statistically significant. However, observe that the pathway from baseline sibling mental health to change in mother mental health was almost twice the magnitude of the pathway from baseline mother mental health to change in sibling mental health. Also note that negative beta value (mother-to-sibling pathway) reflects that the higher the baseline score, the smaller the change score in the other domain, while positive beta value (sibling-to-mother pathway) reflects that the higher the baseline score, the larger the change in the change score of the other domain.

*RQ3: Does baseline parental mental health predict changes in sibling adjustment and does baseline sibling adjustment predict changes in parental mental health?* The BLCS for mother mental health and sibling adjustment is shown in Figure 3. Longitudinal cross-domain associations between mother mental health and sibling adjustment were not statistically significant. Only the self-feedback parameters were statistically significant in this model, as in the previous model.

*RQ4: Are changes in parental mental health associated with changes in sibling mental health and/or adjustment over time?* There was not a significant correlation between the degree of change in mother mental health and sibling mental health after 12 months. Nor was the covariance at baseline statistically significant (Figure 2). A small, significant correlation was found between the degree of change in mother mental health and sibling adjustment. The covariance at baseline was not significant (Figure 3).

A correlation matrix for the three models is presented in Table 3.

**Table 3:** Correlation of measures in the three models.

	∆ SDQ	∆ NAS	Follow-up SDQ	Follow-up NAS	Baseline SDQ	Baseline NAS
∆ SDQ	1.000					
∆NAS	0.288	1.000				
Follow-up SDQ	0.501	0.254	1.000			
Follow-up NAS	0.036	0.448	0.321	1.000		
Baseline SDQ	−0.326	0.027	0.655	0.320	1.000	
Baseline NAS	−0.235	−0.505	0.072	0.545	0.284	1.000

	∆ SCL-90	∆ SDQ	Follow-up SCL-90	Follow-up SDQ	Baseline SCL-90	Baseline SDQ
∆ SCL-90	1.000					
∆ SDQ	−0.060	1.000				
Follow-up SCL-90	0.554	−0.173	1.000			
Follow-up SDQ	0.154	0.507	0.050	1.000		
Baseline SCL-90	−0.286	−0.144	0.640	−0.085	1.000	
Baseline SDQ	0.222	−0.310	0.206	0.662	0.032	1.000

	∆ SCL-90	∆ NAS	Follow-up SCL-90	Follow-up NAS	Baseline SCL90	Baseline NAS
∆ SCL-90	1.000					
∆ NAS	0.061	1.000				
Follow-up SCL-90	0.550	0.146	1.000			
Follow-up NAS	0.215	0.432	0.269	1.000		
Baseline SCL-90	−0.314	0.109	0.620	0.104	1.000	
Baseline NAS	0.139	−0.559	0.107	0.506	−0.009	1.000

SDQ, strengths and difficulties questionnaire; NAS, negative adjustment scale; SCL-90, symptom checklist 90.

## Discussion

We examined potential bidirectional associations between maternal mental health and sibling mental health and adjustment in families of children with a disability. The first research question was if baseline sibling mental health predict changes in sibling adjustment and if baseline sibling adjustment predict changes in sibling mental health? The findings showed significant relationships between sibling adjustment and mental health. Although the bidirectional associations were not statistically significant, the significant baseline covariance suggest that sibling adjustment and mental health are related. The model indicates that while these two domains are interconnected, they remain distinct areas of concern. The significant self-feedback parameters highlight the importance of individual trajectories in each domain.

The second research question was if baseline parental mental health predict changes in sibling mental health, and if baseline sibling mental health predict changes in parental mental health, and the third research questions was if baseline parental mental health predict changes in sibling adjustment and if baseline sibling adjustment predict changes in parental mental health. The findings from our two models showed that the change in maternal mental health was not significantly associated with the baseline levels of mental health or adjustment in siblings, and vice versa. In other words, we did not find a bidirectional relationship between maternal mental health and sibling mental health nor adjustment. This finding contrasts with the transactional model of development (Sameroff, 2009), which posits a reciprocal influence between children and parents over time. According to this model, changes in maternal mental health would be expected to impact sibling mental health and adjustment and vice versa. Our findings, may indicate that these influences are more context-dependent or moderated by other factors. Our findings however align with an earlier bidirectional study (Larrucea-Iruretagoyena and Orue, 2023) which failed to demonstrate a bidirectional relationship between parent and child mental health. Although not statistically significant, we did, however, find a child to mother association that was almost twice the magnitude of the mother to child association in the first model. This is in line with previous studies (Hou et al., 2020; Schulz et al., 2021) which have shown the effect of child mental health on parent mental health to be stronger than the parent mental health on child mental health. The multiple worries combined with limited resources that many parents of children with a disability face (Pinquart, 2018; Wollney et al., 2023) may intensify parental emotional responses to changes in their children’s mental health.

The fourth research question was if changes in parental mental health are associated with changes in sibling mental health and/or adjustment over time. Interestingly, our findings showed that although there was no significant correlation at baseline, changes in maternal mental health and sibling adjustment from baseline to 12 months were correlated, indicating synchronization over time. This finding may reflect that the data were collected as part of an intervention study. Participation in an intervention may have been associated with increased awareness in mothers about siblings’ needs and challenges (both during the intervention and through increased communication after the intervention). Albeit we do not have data to examine this, such awareness may have led to an increased similarity in mothers’ and sibling’ fluctuation in mental health and adjustment. Further, the fact that we found a correlated change in maternal mental health and sibling adjustment, while no such correlated change in maternal mental health and sibling mental health was found, suggests that fluctuations in these families are linked to specific challenges related to childhood disability.

The lack of an identified bidirectional relationship between maternal and sibling mental health and adjustment might be due to small mean changes in all the domains over 12 months. More variance in the data could have led to more significant relationships. All participants in our study participated in a brief intervention 9–12 months before the endpoint of the current study. Previous findings of this intervention have shown improved parent–child relationships up to 6 months after intervention (Fredriksen et al., 2024). However, what is often observed in preventative studies is that early change flattens out over time (Lannes et al., 2021). If we had considered earlier follow-up data than 12 months, we may have observed more variance in the data which in turn could have led to affected bidirectionality findings.

The lack of bidirectionality in our particular sample of mothers and siblings of children with a disability might reflect situation specific fluctuations and trajectories in these families. That is, features that are often observed in families where a child has a disability, such as extra caring responsibilities (Kelada et al., 2022; Pinquart, 2018), less open communication (Haukeland et al., 2022), as well as uncertainty and worry (Wollney et al., 2023), might impact the individual family members above and beyond the mental health and symptoms of the other family members (which would be expected in families with no children with a disability). However, the strain on family processes may vary significantly depending on the specific demands associated with different disabilities. For instance, some disabilities may require intensive, hands-on caregiving that places a continuous strain on family resources and time, affecting both parental availability and sibling adjustment. In such cases, siblings might assume caring responsibilities or experience reduced parental attention, straining family routines and functioning. On the other hand, disabilities with lower caregiving demands may allow for more balanced family interactions. Thus, variability in the type and intensity of caregiving needs is a notable factor in understanding how family processes is affected by strain, potentially shaping the degree of observable bidirectionality in mental health and adjustment.

Another interpretation of the lack of bidirectionality in our models is that while mothers in families of children with a disability typically take on most of the caretaking responsibilities for the affected child (Bögels and Phares, 2008), fathers might play a more significant role in the mental health and adjustment of siblings. Future studies need to include fathers in similar models to explore this hypothesis further. However, studies of general population samples are still inconclusive, meaning, some has also found a non-bidirectional relationship between parent and child mental health, also when including fathers. Besides, in a field that is still emerging with few studies conducted, the fact that the different studies use different measures of parent and child mental health, complicates the comparison and interpretation. Further, inconclusive results between studies might indicate that important moderators are yet to be identified. Thus, future studies should explore potential moderators of bidirectional transactions of mental health and adjustment between parents and children generally, and between mothers and siblings of children with a disability specifically.

The association between changes in maternal mental health and sibling adjustment underscore the importance of including both mothers and siblings in interventions for families of children with disability. This finding is thus an important contribution to the knowledge about families of children with a disability. As a positive change in maternal mental health may reinforce positive change in siblings, or the other way around, this finding can facilitate the development of well-aimed interventions and allocation of resources in family-centered care. Further, the findings may suggest that fluctuations in family mental health are linked to specific challenges related to childhood disability. This underscores that well-aimed support interventions for families of children with a disability, such as practical or financial assistance in the home or at school, can benefit not only the affected child or their parents, but the entire family system.

### Strengths and limitations

A major limitation of this study is the lack of data from fathers. The majority of studies on familial mental health dynamics have focused on mothers (Risi et al., 2021). Through global social and demographic changes over the last decades, fathers are today seen as actively, and independently, impacting child development (Cabrera et al., 2018). As fathers have come to be more involved in caregiving, studies on paternity and mental health have increased accordingly (e.g., Foster et al., 2022; Sweeney and MacBeth, 2016). However, our knowledge on paternal impact on child mental health is still small compared to our knowledge about maternal factors. We originally aimed to run analyses of both mother-sibling and father-sibling dyads. Father scores however, unfortunately had to be excluded due to low response rate at 12-month follow-up. This exclusion leaves a gap in our understanding of father-sibling relationships in families with children diagnosed with a disability. To improve data collection in future studies, strategies to minimize attrition and ensure higher father response rates in later waves should be implemented.

A further limitation is the relatively modest sample size for BLCS models. This may have made it hard to detect possible bidirectional effects. The previous study finding significant bidirectional associations in a cross-lagged model (Hou et al., 2020) had a considerable larger sample size (*N* = 1,644), whereas the previous studies finding only a child driven effect or no significant effects (Larrucea-Iruretagoyena and Orue, 2023; Schulz et al., 2021) had more modest sample sizes (i.e., *N* ≤ 497). However, in terms of effect size, the standardized regression (beta) coefficients reported in the mentioned studies were, respectively, 0.06 for mother-to-adolescent effects and 0.15 for adolescent-to-mother effect (Hou et al., 2020), and 0.05 for mother-to-adolescent effect (non-significant) and 0.12 for adolescent-to-mother effect (Schulz et al., 2021). This indicates strong adolescent-to-parent effects and medium parent-to-adolescent effects in both studies, as suggested by Orth et al. (2024). This is a similar pattern to what we found in our study, with child-to-parent coefficients being stronger in both models. In the mother mental health–sibling mental health model, the relative difference between effects was similar to the findings of the mentioned studies.

Another limitation of our study is that we only directly analyzed the observed variables, without imposing any model restrictions. The model used is simply a reformulation of the covariance matrix, so while it employs the concept of latent change scores as defined by McArdle and Nesselroade (2014, p. 89), this approach is formally equivalent to calculating the difference between two observed scores (post-test and pre-test), with this change treated as a latent variable. Having a just-identified model does not invalidate it per se; this structure is inherent to the model and has been revisited in work by Kievit et al. (2018). However, its foundations stem from earlier works, initially defined in McArdle and Nesselroade (1994). To address the limitations of a just-identified model (and its lack of model fit), one approach would be to (a) add more repeated measurements and (b) incorporate different raters at each time point (e.g., mother and father) or different components of the scale (e.g., item-level data). Both adjustments would create a more robust model, allowing for traditional model fit testing (e.g., CFI, TLI, RMSEA). However, in terms of multiple raters, this was hindered by the high attrition of father respondents (only 26 fathers reported their mental health status at the 12-month follow-up). For item-level analysis, from which latent change factors could be derived, the main issue was the small ratio between the number of items per scale and the sample size (i.e., 90 items for the SCL-90, 25 items for the SDQ, and 16 items for the NAS). For instance, a dyadic model at the item level for the SCL-90 and SDQ would involve 180 + 50 items, totaling 230 items for pre- and post-testing, not including their respective factor loadings and thresholds, which would be unfeasible with a sample size of fewer than 300 participants. Other limitations of the study are the high percentage of White-European American families with highly educated mothers, causing an under-representation of families from other ethnicities and mothers of lower education levels.

## Summary and conclusion

Our study examined the potential bidirectional associations between maternal mental health and sibling mental health and adjustment. The aim was to explore the extent to which baseline levels in mother and sibling measures could predict the change in mother and sibling scores over a 12-month period. The findings demonstrated a correlated change between maternal mental health and sibling adjustment after 12 months, suggesting a synchronization over time, possibly influenced by shared family stressors related to childhood disability. Changes in maternal mental health were not significantly associated with baseline levels of mental health or adjustment in siblings or vice versa, indicating no bidirectional relationship. However, we observed a child to mother association that was nearly twice the magnitude of the mother to child association, aligning with prior studies. Overall, our study contributes to the growing body of research on parent–child mental health relationships, emphasizing the need for further investigation into potential moderators that could explain variations in these associations.

The key takeaway message from this study is that while changes in maternal mental health and sibling adjustment are correlated, they do not operate in a strictly bidirectional way. This finding supports a family-centeredness in pediatric patient care, while at the same time underscores the need for a broader understanding of familial mental health dynamics, considering a wider range of factors. Future research should strive for more comprehensive data collection, to include fathers in models of bidirectionality, to understand family dynamics better. Future studies should also aim to identify moderators that might clarify these complex interfamilial relationships.

## Data Availability

The raw data supporting the conclusions of this article will be made available by the authors, without undue reservation.

## References

[ref1] Anaby D. Hand C. Bradley L. DiRezze B. Forhan M. DiGiacomo A.. (2013). The effect of the environment on participation of children and youth with disabilities: a scoping review. Disabil. Rehabil. 35, 1589–1598. doi: 10.3109/09638288.2012.748840, PMID: 23350759

[ref2] Asparouhov T. (2005). Sampling weights in latent variable modeling. Struct. Equ. Model. Multidiscip. J. 12, 411–434. doi: 10.1207/s15328007sem1203_4

[ref3] Asparouhov T. (2006). General multi-level modeling with sampling weights. Commun. Stat. Theory Methods 35, 439–460. doi: 10.1080/03610920500476598

[ref4] Bögels S. Phares V. (2008). Fathers’ role in the etiology, prevention and treatment of child anxiety: a review and new model. Clin. Psychol. Rev. 28, 539–558. doi: 10.1016/j.cpr.2007.07.011, PMID: 17854963

[ref5] Cabrera N. J. Volling B. L. Barr R. (2018). Fathers are parents, too! Widening the Lens on parenting for Children’s development. Child Dev. Perspect. 12, 152–157. doi: 10.1111/cdep.12275

[ref7001] Derogatis L. R. (2010). SCL-90-R®. Symptom Checklist-90-R. Norsk versjon. Manual for administrering og skåring [SCL-90-R®. Symptom Checklist-90-R. Norwegian version. Manual for administration and scoring]. Stockholm: NCS Pearson, Inc.

[ref6] Enders C. K. (2010). Applied missing data analysis, methodology in the social sciences. New York: Guilford Press.

[ref7] Enders C. K. Bandalos D. L. (2001). The relative performance of full information maximum likelihood estimation for missing data in structural equation models. Struct. Equ. Model. 8, 430–457. doi: 10.1207/S15328007SEM0803_5

[ref8] Engelhard C. Hishinuma E. Rehuher D. (2022). The impact of maternal depression on child mental health treatment and models for integrating care: a systematic review. Arch. Womens Ment. Health 25, 1041–1065. doi: 10.1007/s00737-022-01272-2, PMID: 36327004

[ref9] Fjermestad K. W. Silverman W. K. Vatne T. M. (2020). Group intervention for siblings and parents of children with chronic disorders (SIBS-RCT): study protocol for a randomized controlled trial. Trials 21:851. doi: 10.1186/s13063-020-04781-6, PMID: 33054825 PMC7556945

[ref10] Foster D. Rodrigues M. Somir I. Aziz T. Patel R. Ragunathan S.. (2022). Paternal positivity and child mental health: a Meta-analysis. J. Child Fam. Stud. 31, 2556–2570. doi: 10.1007/s10826-022-02361-7

[ref11] Fredriksen T. Vatne T. M. Haukeland Y. B. Czajkowski N. O. Wakefield C. E. Fjermestad K. W. (2024). Evaluation of siblings’ perceived relationship outcomes with their parents in an open trial of the SIBS intervention for children with chronic disorders. J. Child Fam. Stud. 33, 2271–2285. doi: 10.1007/s10826-024-02824-z

[ref12] Gau S. S.-F. Chou M.-C. Chiang H.-L. Lee J.-C. Wong C.-C. Chou W.-J.. (2012). Parental adjustment, marital relationship, and family function in families of children with autism. Res. Autism Spectr. Disord. 6, 263–270. doi: 10.1016/j.rasd.2011.05.007

[ref13] Goodman R. (1997). The strengths and difficulties questionnaire: a research note. J. Child Psychol. Psychiatry 38, 581–586. doi: 10.1111/j.1469-7610.1997.tb01545.x, PMID: 9255702

[ref14] Goodman R. (2001). Psychometric properties of the strengths and difficulties questionnaire. J. Am. Acad. Child Adolesc. Psychiatry 40, 1337–1345. doi: 10.1097/00004583-200111000-00015, PMID: 11699809

[ref15] Haukeland Y. B. Fjermestad K. W. Mossige S. Vatne T. M. (2015). Emotional experiences among siblings of children with rare disorders. J. Pediatr. Psychol. 40, 712–720. doi: 10.1093/jpepsy/jsv022, PMID: 25817880

[ref16] Haukeland Y. B. Fjermestad K. W. Mossige S. Vatne T. M. (2022). Parent-child communication about emotions during SIBS: a joint intervention for siblings and parents of children with chronic disorders. Nord. Psychol. 74, 205–221. doi: 10.1080/19012276.2021.1986850

[ref17] Hou J. Chen Z. Guo F. (2020). The transactional relationship between parental and adolescent depressive symptoms: the mediating effect of Nurturant–involved parenting. Int. J. Environ. Res. Public Health 17:8240. doi: 10.3390/ijerph17218240, PMID: 33171873 PMC7664705

[ref18] Incledon E. Williams L. Hazell T. Heard T. R. Flowers A. Hiscock H. (2015). A review of factors associated with mental health in siblings of children with chronic illness. J. Child Health Care 19, 182–194. doi: 10.1177/1367493513503584, PMID: 24270987

[ref19] Kelada L. Wakefield C. E. Drew D. Ooi C. Y. Palmer E. E. Bye A.. (2022). Siblings of young people with chronic illness: caring responsibilities and psychosocial functioning. J. Child Health Care 26, 581–596. doi: 10.1177/13674935211033466, PMID: 34271837

[ref20] Kievit R. A. Brandmaier A. M. Ziegler G. van Harmelen A.-L. de Mooij S. M. M. Moutoussis M.. (2018). Developmental cognitive neuroscience using latent change score models: a tutorial and applications. Dev. Cogn. Neurosci. 33, 99–117. doi: 10.1016/j.dcn.2017.11.007, PMID: 29325701 PMC6614039

[ref21] Kirchhofer S. M. Fredriksen T. Orm S. Botta M. Zahl E. Cogo-Moreira H.. (2024). A randomized controlled group intervention trial for siblings of children with chronic disorders. Oslo: Manuscr. Submitt. Publ. Dep. Psychol. Univ.

[ref22] Kirchhofer S. M. Orm S. Haukeland Y. B. Fredriksen T. Wakefield C. E. Fjermestad K. W. (2022). A systematic review of social support for siblings of children with neurodevelopmental disorders. Res. Dev. Disabil. 126:104234. doi: 10.1016/j.ridd.2022.104234, PMID: 35468570

[ref23] Kuhlthau K. A. Ames S. G. Ware A. Hoover C. G. Wells N. Shelton C. (2022). Research on family health and children and youth with special health care needs. Acad. Pediatr 22, S22–S27. doi: 10.1016/j.acap.2021.07.019, PMID: 35248244

[ref24] Lannes A. Bui E. Arnaud C. Raynaud J.-P. Revet A. (2021). Preventive interventions in offspring of parents with mental illness: a systematic review and meta-analysis of randomized controlled trials. Psychol. Med. 51, 2321–2336. doi: 10.1017/S0033291721003366, PMID: 34435556

[ref25] Larrucea-Iruretagoyena M. Orue I. (2023). Bidirectional relationships between parental anxiety, internalizing symptoms, and peer victimization and aggression among early adolescents. J. Early Adolesc. 44, 96–118. doi: 10.1177/02724316231160146, PMID: 39659294

[ref26] Little R. J. A. Rubin D. B. (2002). Statistical analysis with missing data. 2nd Edn Wiley series in probability and statistics. Hoboken (N.J.): Wiley.

[ref27] Lobato D. J. Kao B. T. (2002). Integrated sibling-parent group intervention to improve sibling knowledge and adjustment to chronic illness and disability. J. Pediatr. Psychol. 27, 711–716. doi: 10.1093/jpepsy/27.8.711, PMID: 12403861

[ref28] Marquis S. M. McGrail K. Hayes M. V. (2019). A population-level study of the mental health of siblings of children who have a developmental disability. SSM Popul. Health 8:100441. doi: 10.1016/j.ssmph.2019.100441, PMID: 31334325 PMC6617296

[ref29] Martinez B. Pechlivanoglou P. Meng D. Traubici B. Mahood Q. Korczak D.. (2022). Clinical health outcomes of siblings of children with chronic conditions: a systematic review and Meta-analysis. J. Pediatr. 250, 83–92.e8. doi: 10.1016/j.jpeds.2022.07.002, PMID: 35810772

[ref30] McArdle J. J. Nesselroade J. R. (1994). “Using multivariate data to structure developmental change” in Life-span Developmental Psychology. eds. Cohen S. H. Reese H. W. (United Kingdom: Routledge), 223–267.

[ref31] McArdle J. J. Nesselroade J. R. (2014). Longitudinal data analysis using structural equation models. Washington: American Psychological Association.

[ref32] Muthén L. K. Muthén B. (2017). Mplus user’s guide: Statistical analysis with latent variables, user’s guide. Los Angeles, CA: Muthén & Muthén.

[ref33] Orm S. Vatne T. Haukeland Y. B. Silverman W. K. Fjermestad K. (2021). The validity of a measure of adjustment in siblings of children with developmental and physical disabilities: a brief report. Dev. Neurorehabil. 24, 355–358. doi: 10.1080/17518423.2020.1869338, PMID: 33393399

[ref34] Orth U. Meier L. L. Bühler J. L. Dapp L. C. Krauss S. Messerli D.. (2024). Effect size guidelines for cross-lagged effects. Psychol. Methods 29, 421–433. doi: 10.1037/met0000499, PMID: 35737548

[ref35] Ortuño-Sierra J. Sebastián-Enesco C. Pérez-Albéniz A. Lucas-Molina B. Fonseca-Pedrero E. (2022). Spanish normative data of the strengths and difficulties questionnaire in a community-based sample of adolescents. Int. J. Clin. Health Psychol. 22:100328. doi: 10.1016/j.ijchp.2022.100328, PMID: 36111263 PMC9442435

[ref36] Pettoello-Mantovani M. Campanozzi A. Maiuri L. Giardino I. (2009). Family-oriented and family-centered care in pediatrics. Ital. J. Pediatr. 35:12. doi: 10.1186/1824-7288-35-12, PMID: 19490603 PMC2691736

[ref37] Pinquart M. (2018). Parenting stress in caregivers of children with chronic physical condition-a meta-analysis. Stress. Health 34, 197–207. doi: 10.1002/smi.2780, PMID: 28834111

[ref38] Ramlall I. (2016). Model identification, in: Applied structural equation modelling for researchers and practitioners. Leeds: Emerald Group Publishing Limited, 51–56.

[ref39] Risi A. Pickard J. A. Bird A. L. (2021). The implications of parent mental health and wellbeing for parent-child attachment: a systematic review. PLoS One 16:e0260891. doi: 10.1371/journal.pone.0260891, PMID: 34914730 PMC8675743

[ref40] Sameroff A. J. (2009). The transactional model of development: How children and contexts shape each other. Washington: American Psychological Association.

[ref41] Scherer N. Verhey I. Kuper H. (2019). Depression and anxiety in parents of children with intellectual and developmental disabilities: a systematic review and meta-analysis. PLoS One 14:e0219888. doi: 10.1371/journal.pone.0219888, PMID: 31361768 PMC6667144

[ref42] Schulz S. Nelemans S. A. Oldehinkel A. J. Meeus W. Branje S. (2021). Examining intergenerational transmission of psychopathology: associations between parental and adolescent internalizing and externalizing symptoms across adolescence. Dev. Psychol. 57, 269–283. doi: 10.1037/dev0001144, PMID: 33346677

[ref7002] Siqveland J. Moum T. Leiknes K. A. (2016). Måleegenskaper ved den norske versjonen av Symptom Checklist 90 Revidert (SCL-90-R). [Assessment of psychometric properties of the Norwegian version of the Symptom Checklist 90 Revised (SCL-90-R)]. Oslo: Folkehelseinstituttet.

[ref43] Steyer R. Eid M. Schwenkmezger P. (1997). Modeling true intraindividual change: true change as a latent variable. Methods Psychol. Res. 2, 21–33.

[ref44] Stoneman Z. (2001). Supporting positive sibling relationships during childhood. Ment. Retard. Dev. Disabil. Res. Rev. 7, 134–142. doi: 10.1002/mrdd.1019, PMID: 11389569

[ref45] Sweeney S. MacBeth A. (2016). The effects of paternal depression on child and adolescent outcomes: a systematic review. J. Affect. Disord. 205, 44–59. doi: 10.1016/j.jad.2016.05.073, PMID: 27414953

[ref46] Theunissen M. H. C. de Wolff M. S. Eekhout I. Mieloo C. L. Stone L. L. Reijneveld S. A. (2022). The strengths and difficulties questionnaire parent form: Dutch norms and validity. BMC Pediatr. 22, 202–209. doi: 10.1186/s12887-022-03274-6, PMID: 35413892 PMC9004049

[ref47] Vugteveen J. de Bildt A. Timmerman M. E. (2022). Normative data for the self-reported and parent-reported strengths and difficulties questionnaire (SDQ) for ages 12–17. Child Adolesc. Psychiatry Ment. Health 16:5. doi: 10.1186/s13034-021-00437-8, PMID: 35042556 PMC8764849

[ref48] Wolff B. Franco V. R. Magiati I. Cooper M. N. Roberts R. Skoss R.. (2023). Individual-level risk and resilience factors associated with mental health in siblings of individuals with neurodevelopmental conditions: a network analysis. Dev. Neuropsychol. 48, 112–134. doi: 10.1080/87565641.2023.2190119, PMID: 36942456

[ref49] Wollney E. N. Bylund C. L. Kastrinos A. L. Campbell-Salome G. Sae-Hau M. Weiss E. S.. (2023). Understanding parents uncertainty sources and management strategies while caring for a child diagnosed with a hematologic cancer. PEC Innov. 3:100198. doi: 10.1016/j.pecinn.2023.100198, PMID: 37662692 PMC10468798

[ref50] World Health Organization (2011). World report on disability. Geneva: World Health Organization.

[ref51] World Health Organization (2014). Integrating the response to mental disorders and other chronic diseases in health care systems. Geneva: World Health Organization.

[ref53] Zahl E. Willemen A. M. Fredriksen T. Kirchhofer S. M. Vatne T. M. Orm S.. (2024). Mental health in mothers and fathers of children with chronic disorders. PEC Innov. 5:100331. doi: 10.1016/j.pecinn.2024.100331, PMID: 39252881 PMC11381899

