# Supplementary material for: Mental health dynamics between mothers and siblings of children with disabilities
Source: Front Psychol. 2024 Dec 24;15:1501343. doi: 10.3389/fpsyg.2024.1501343 (PMC11703835; doi:10.3389/fpsyg.2024.1501343)
Supplement: Supplementary file 1 [file Table_1.pdf]

Supplementary tables

Table S1

Logistic regression model for data completeness of siblings' outcome at 12 months

	B	*SE*	Wald	*df*	*p*	Exp(B)
Sibling age	0,055	0,090	0,380	1	0,537	1,057
Sibling sex	0,365	0,367	0,988	1	0,320	1,440
Mother age	0,045	0,040	1,266	1	0,260	1,046
Mother education level	0,095	0,265	0,128	1	0,720	1,100
Mother employment status	0,040	0,110	0,132	1	0,717	1,041
Attention Deficit Hyperactivity Disorder	-0,015	0,477	0,001	1	0,975	0,985
Tourette Syndrome	0,445	0,594	0,561	1	0,454	1,560
Asperger Syndrome	-0,218	0,650	0,113	1	0,737	0,804
Autism	-0,406	0,627	0,419	1	0,517	0,666
Specific developmental disorders	-0,521	1,116	0,218	1	0,641	0,594
Emotional or conduct disorders	0,373	0,492	0,576	1	0,448	1,453
Eating Disorders	0,028	0,809	0,001	1	0,973	1,028
Intellectual Disability	0,314	0,626	0,251	1	0,616	1,369
Down Syndrome	-0,978	0,915	1,143	1	0,285	0,376
Rare Disorders	0,401	0,611	0,431	1	0,511	1,494
Somatic Disorders	-1,755	1,063	2,726	1	0,099	0,173

Note. Diagnoses were parent reported and cross checked with clinic registry.

Table S2

Logistic regression model for data completeness of mothers' outcome at 12 months

	B	*SE*	Wald	*df*	*p*	Exp(B)
Sibling age	0,033	0,079	0,170	1	0,680	1,033
Sibling sex	-0,211	0,312	0,457	1	0,499	0,810
Mother age	0,026	0,033	0,599	1	0,439	1,026
Mother education level	0,035	0,229	0,024	1	0,878	1,036
Mother employment status	-0,053	0,098	0,288	1	0,592	0,949
Attention Deficit Hyperactivity Disorder	0,204	0,409	0,248	1	0,618	1,226
Tourette Syndrome	0,038	0,552	0,005	1	0,946	1,038
Asperger Syndrome	-0,712	0,596	1,427	1	0,232	0,491
Autism	-0,456	0,531	0,737	1	0,390	0,634
Specific developmental disorders	-0,257	0,845	0,093	1	0,761	0,773
Emotional or conduct disorders	0,204	0,457	0,199	1	0,656	1,226
Eating Disorders	-0,313	0,775	0,163	1	0,686	0,731
Intellectual Disability	0,446	0,502	0,788	1	0,375	1,562
Down Syndrome	-0,352	0,673	0,273	1	0,601	0,704
Rare Disorders	0,352	0,514	0,470	1	0,493	1,422
Somatic Disorders	-0,167	0,536	0,097	1	0,755	0,846

Note. Diagnoses were parent reported and cross checked with clinic registry.
